# Psychological benefits 2 and 4 weeks after a single treatment with near infrared light to the forehead: a pilot study of 10 patients with major depression and anxiety

**DOI:** 10.1186/1744-9081-5-46

**Published:** 2009-12-08

**Authors:** Fredric Schiffer, Andrea L Johnston, Caitlin Ravichandran, Ann Polcari, Martin H Teicher, Robert H Webb, Michael R Hamblin

**Affiliations:** 1 The Department of Psychiatry, Harvard Medical School and the Developmental Biopsychiatry Research Program, McLean Hospital, 115 Mill Street Belmont, MA 02478 USA; 2 The Department of Psychiatry, Harvard Medical School and the Laboratory for Psychiatric Biostatistics, McLean Hospital, 115 Mill Street Belmont, MA 02478 USA; 3 Wellman Center for Photomedicine, Massachusetts General Hospital, 40 Blossom Street, Boston, MA 02114, USA; 4 Department of Dermatology, Harvard Medical School, Boston, MA 02115, USA; 5 Harvard-MIT Division of Health Sciences and Technology, Cambridge, MA 02139, USA

## Abstract

**Background:**

Many studies have reported beneficial effects from the application of near-infrared (NIR) light photobiomodulation (PBM) to the body, and one group has reported beneficial effects applying it to the brain in stroke patients. We have reported that the measurement of a patient's left and right hemispheric emotional valence (HEV) may clarify data and guide lateralized treatments. We sought to test whether a NIR treatment could 1. improve the psychological status of patients, 2. show a relationship between immediate psychological improvements when HEV was taken into account, and 3. show an increase in frontal pole regional cerebral blood flow (rCBF), and 4. be applied without side effects.

**Methods:**

We gave 10 patients, (5 M/5 F) with major depression, including 9 with anxiety, 7 with a past history of substance abuse (6 with an opiate abuse and 1 with an alcohol abuse history), and 3 with post traumatic stress disorder, a baseline standard diagnostic interview, a Hamilton Depression Rating Scale (HAM-D), a Hamilton Anxiety Rating Scale (HAM-A), and a Positive and Negative Affect Scale (PANAS). We then gave four 4-minute treatments in a random order: NIR to left forehead at F3, to right forehead at F4, and placebo treatments (light off) at the same sites. Immediately following each treatment we repeated the PANAS, and at 2-weeks and at 4-weeks post treatment we repeated all 3 rating scales. During all treatments we recorded total hemoglobin (cHb), as a measure of rCBF with a commercial NIR spectroscopy device over the left and the right frontal poles of the brain.

**Results:**

At 2-weeks post treatment 6 of 10 patients had a remission (a score ≤ 10) on the HAM-D and 7 of 10 achieved this on the HAM-A. Patients experienced highly significant reductions in both HAM-D and HAM-A scores following treatment, with the greatest reductions occurring at 2 weeks. Mean rCBF across hemispheres increased from 0.011 units in the off condition to 0.043 units in the on condition, for a difference of 0.032 (95% CI: -0.016, 0.080) units, though this result did not reach statistical significance. Immediately after treatment the PANAS improved to a significantly greater extent with NIR "on" relative to NIR "off" when a hemisphere with more positive HEV was treated than when one with more negative HEV was treated. We observed no side effects.

**Conclusion:**

This small feasibility study suggests that NIR-PBM may have utility for the treatment of depression and other psychiatric disorders and that double blind randomized placebo-controlled trials are indicated.

**Trial registration:**

ClinicalTrials.gov Identifier: NCT00961454

## Background

The National Comorbidity Survey [1] reported that 46% of men and 58% of women were found to have suffered in their lifetime at least a two week period in which they experienced a persistent depressed mood. Major depression disorder (MDD) has a lifetime prevalence of about 16% [2], and it is estimated that by 2020, it will be the second greatest contributor to the impairment of global health [3]. A recent Australian survey reported that anxiety disorders were the most common mental disorder with a lifetime prevalence of 26% [4]. We present our findings from an open study of a novel therapy for these prevalent, deleterious conditions.

Photobiomodulation (PBM), also known as low level laser therapy (LLLT), is the application of phototherapy, often from a red or near-infrared laser, or from a non-coherent light source, such as a light emitting diode (LED). It been reported in over a thousand scientific publications to have therapeutic efficacy for a wide range of disorders in humans without any observed harmful effects. PBM has been demonstrated in cell culture to increase mitochondrial respiration [5], increase ATP synthesis [5-7], upregulate expression of reactive oxygen species [8], modulate the expression of 111 genes in a cDNA microarry study [9], and increase nerve cell proliferation and migration [10]. PBM has been tested in animals to facilitate wound healing [11], improve inflammatory arthritis [12], promote the process of skeletal muscle regeneration [13], and reduce infarct size in ischemic heart muscle by 50 to 70% in an induced experimental model in rats and dogs [14]. Transcranial PBM, using near-infrared light which penetrates the scalp and skull, can significantly reduce damage from experimentally induced stroke in rats [10] and rabbits [15], can improve the memory performance of middle aged mice [16], and has been shown to reduce damage from acute stroke in humans [17,18].

Several studies have suggested that depression is associated with abnormalities of frontal activation reflected in abnormalities in frontal regional cerebral blood flow (rCBF) [19-21]. PBM has induced increases in blood circulation in the hands of patients with Raynaud's phenomenon [22,23], in skin flaps [24], and in healthy skin [25]. We sought to examine whether transcranial PBM might alter pre-frontal rCBF as well as whether it can affect the emotional status of patients with major depression with anxiety. We see this small feasibility study as the first of a series of experiments to explore eventually whether PBM might be useful as a safe and effective treatment for psychological disorders, and whether any observed improvement might have a relationship with alterations in our measurements of rCBF and hemispheric emotional valence (HEV), the tendency for one cerebral hemisphere (either left or right) to have, as a trait, a more positive psychological disposition than the other.

Several treatments for depression, such as transcranial magnetic stimulation [26], deep brain stimulation [27], and electro-convulsive therapy [28], transcranial direct current stimulation [29,30], apply energy to the brain and have been effective in the treatment of major depressive disorder (MDD) even though their mechanisms remain uncertain. We believe this is the first study of transcranial PBM, which also applies energy, as a treatment for any psychological illness.

## Methods

The protocol was approved by the Massachusetts General Hospital (MGH) Institutional Review Board and was conducted in accord with the principles of the Helsinki Declaration. We studied 10 right-handed patients, described in Tables 1, 2, and 3, who were recruited through advertisements posted on the internet and at a substance abuse clinic. Among those with a history of substance abuse, 6 had a past history of opiate dependence, and 1 had a past history of alcohol dependence. Enrollment was made without regard to gender or ethnicity. Inclusion criterion allowed for patients receiving mental health care if they had not altered their treatment during the month preceding the study. At enrollment we asked that they try, but not be required, to maintain their usual treatment until the study's conclusion. All patients complied with this request, and no patient altered their usual treatment for the duration of the 4-week study. We excluded patients who were not right handed, not between the ages of 18 and 60 or failed to meet the structured clinical interview for DSM-IV (SCID) criteria for MDD. We also excluded any person with a past history of a psychotic disorder, a substance abuse disorder that had been active within the 6 months prior to the study, a history of violent behavior, a history of a past suicide gesture or attempt, a history of current suicidal ideation, a history of a neurological condition (e.g. epilepsy, traumatic brain injury, stroke), pregnancy, or a current acute or chronic medical condition. We would have excluded any person whom we judged to have an impaired decision-making capacity. Prior to enrollment, we obtained informed consent according to the existing policies at MGH. No patient, once enrolled, failed to complete the study.

**Table 1:** Demographics: Age, gender, and SCID diagnosis

			SCID Diagnosis			
			
Subject #	Age	Gender	Major Depression	Anxiety Disorder	PTSD	Subs abuse history
1	38	M	+	+		
2	26	F	+			
3	27	M	+	+	+	opiate
4	42	M	+	+		alcohol
5	40	F	+	+		opiate
6	34	M	+	+	+	opiate
7	35	M	+	+		opiate
8	25	F	+	+	+	opiate
9	46	F	+	+		
10	38	F	+	+		opiate

Mean ± SD	35 ± 7					

N	10	5 M	10	9	3	7

**Table 2:** Baseline measurements of outcome measures and hemispheric emotional valence.

Subject #	Initial HAMD	Initial HAMA	Initial PANAS	HEV value	HEV category
1	19	16	11	-2	Right Negative
2	14	6	1.5	-1.5	Right Negative
3	26.5	19	11	-1	Right Negative
4	26	26	6	2	Left Negative
5	27	22	6.5	4	Left Negative
6	32	38	-5	11	Left Negative
7	11	16	3	26	Left Negative
8	33	48	-4	7	Left Negative
9	14	15	7	-1	Right Negative
10	36	24	2	-1	Right Negative

Mean ± SD	23.9 ± 8.8	23.0 ± 12.2	3.9 ± 5.5	4.35 ± 8.7	

N					5R/5L

**Table 3:** Unaltered treatments before and during the study period.

	Treatment				
	
Subject #	ssri or snri	benzodiazepine	buphenorphine	methadone	psychotherapy
1	+				
2	+				
3	+	+		+	+
4	+				
5	+	+	+		+
6	+	+	+		+
7			+		+
8			+		
9	+				
10	+	+	+		

N	8	4	5	1	4

### Instruments

#### Photobiomodulation with near infrared light

The treatment consisted of applying PBM in the form of a light emitting diode (LED) array (Marubeni America Corp, Santa Clara, CA) with a peak wavelength of 810 nm with a full width half maximum of 40 nm, delivering an irradiance of 250 mW/cm^2 ^when applied at 4 mm from the skin. The treatment consisted of exposure to the light for 4 minutes (total delivered fluence per site of 60 J/cm^2^) at each of 2 sites on the forehead that correspond to the 10-20 EEG sites, F3, and F4. Based on a penetration of 3.7% of the light to the dura, we calculated that 2.1 J/cm^2 ^was delivered to each of the treated areas of the brain. The level of light exposure at the skin was well below the irradiance allowed by the ANSI standard of 320 mW/cm^2^. Based on that standard, we conclude that the level of light exposure either to the skin (power density of 250 mW/cm^2 ^and total fluence of 60 J/cm^2^) and to the surface of the brain (power density of 9.5 mW/cm^2 ^and total fluence of 2.1 J/cm^2^) to each of the 2 treated areas of the forehead poses no significant risk as discussed above. Subjects wore protective eyewear even though the physician administering the PBM was careful to not shine the light in or near the eyes. The output of our device is at least 5 times less than the PhotoThera laser device (personal communication, Luis DeTaboada, PhotoThera Inc, Carlsbad, CA) that was used without observed side-effects in stroke patients [17], and was found in a study of the rat brains exposed to light to cause no observable behavioral or cellular alterations [31]. In the human stroke study, the patients' heads were shaved and they were treated at 20 sites around the entire head. Subjects were not shaved in the present study as light was applied only to the forehead.

The rationale for the optical parameters were as follows: The wavelength of 810-nm is optimum for light penetration of living tissue due to minimization of absorption by all three major tissue chromophores, hemoglobin, melanin and water. Moreover this wavelength has been shown to be effectively absorbed by mitochondria that are believed to be responsible for the biological effects of photobiomodulation. The energy density (60 J/cm^2^) was chosen with reference to other published studies reporting transcranial laser for stroke in humans and knowledge about the optical properties of human tissue as discussed in the text. The power density (250 mW/cm^2^) was chosen to be safe and avoid heating of the skin.

#### Near Infra-red spectroscopy (NIRS) for the measurement of total oxy and deoxy-hemoglobin (cHb) in the left and right frontal poles

We measured cHb in left and right frontal poles by NIRS, using an INVOS system (Somanetics, Troy, MI) http://www.somanetics.com/invos-system, modified by Somanetics to provide cHb, which we believe to be our best reflection of rCBF, in addition to the device's usual oxygen saturation output. The Somanetics device is FDA approved, is commercially available, and is used throughout the world in hospital settings to monitor cerebral perfusion. It poses no harm or discomfort to subjects, yet is convenient, and allows the subject to have relatively free movement. This device can be used to monitor cHb in the left and right frontal poles during PBM. Since our PBM uses continuous wave emission, its light is not detected by this NIRS device because it has a proprietary mechanism for excluding continuous light so that ambient light does not contaminate the device's pulsed photon emitter/detector.

### Affect measures

We evaluated the psychological state of patients with the following instruments: Standard Clinical Diagnostic Interview (SCID) [32], a Hamilton Depression Rating Scale 21-item (HAM-D) [33], a Hamilton Anxiety Rating Scale (HAM-A) [34], and a Positive and Negative Affect Scale (PANAS) [35]. We searched for side effects of the treatment with a form we constructed with both open-ended questions and a physical and psychological symptom check list.

### Determination of hemispheric emotional valence

Lateral visual field stimulation (LVFS), a simple test, consisting of blocking one visual field so that the patient is looking exclusively out of the left or right lateral visual field, at a photograph of a man or woman with an ambiguous emotional expression. The subject does so for one minute then his affects are rated on an abbreviated PANAS scale [36-39]. LVFS has been shown to alter hemispheric activation by BOLD fMRI [40]. HEV determined by LVFS has predicted in two independent studies the outcomes to a 2-week course of left-sided rTMS for depression [41,42]. We have suggested that HEV might be used to guide the application lateral treatments to the brain and aid in the evaluation of experimental data [39].

From the subjects' left and right-sided LVFS scores on the PANAS we derived the patients' hemispheric emotional valence (HEV). To determine the patient's baseline HEV, before any treatments, we used the PANAS scores recorded one minute after his or her looking out the right visual field (RVF) and that recorded one minute after his looking out the left visual field (LVF). We use this order of testing for all patients. We used the difference between the PANAS scores during the LVF - the RVF to determine the HEV, for which we obtained an individual score. Since the LVF is thought to relate to the right hemisphere, when LVF-RVF was positive (right hemisphere had more positive affect), we assigned a left negative HEV, and when it was negative, a right negative HEV.

### Study design and procedures

**Figure 1:**
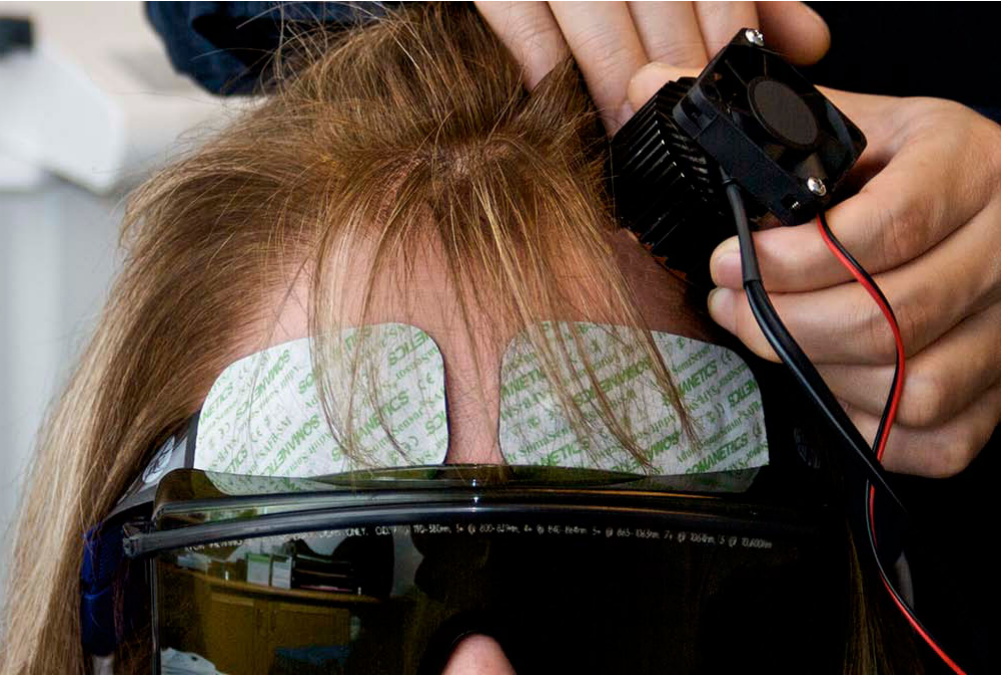
**Near infrared treatment**. The NIR LED array is a few millimeters from the skin beneath a heat sink and cooling fan at F3. Somanetics "SomaSensors" with NIR photon emitters and detectors are applied just above each eyebrow to measure left- and right-sided total hemoglobin.

Each of the 10 patients who met our criteria by a phone interview came to our laboratory and gave written informed consent according to approved protocol. Then each was given a SCID, followed by a baseline HAM-D, HAM-A, PANAS, and LVFS. Subjects were not drug tested, but patients with a history of opiate abuse had been in a stable treatment program for at lease 6-months, and each was believed by their prescribing psychiatrist to have abstained from illicit drugs for this period. Each subject was then connected to our NIRS device by having 5 by 2.5 cm^2 ^adhesive pads containing a photon emitter and detectors attached to each side of the forehead immediately over the eyebrows, as shown in Figure 1.

The NIRS device collected data continuously at 1-second intervals throughout the study. Event marks indicated the beginning and end of each baseline or treatment period. Data were zeroed at the beginning of each period. A researcher, blind to the treatments, administered the PANAS scales at baseline and immediately after each of the 4 treatments. A different researcher administered the HAM-D and the HAM-A at baseline and at 2- and 4-weeks post treatment. He was not blinded because all patients received active treatments during the treatment day. In random order, the patient was given the first of 4 interventions, consisting of: A). NIR "on" for 4 minutes at F3 (of the 10-20 EEG system), left forehead approximately over the left dorsolateral prefrontal cortex, B). NIR "on" for 4 minutes at F4, C). NIR "off" with the NIR device held at F3 for 4 minutes, as shown in Figure 1). The same as intervention #C but at F4. Thus, we had two active treatments and two placebo treatments. A cooling fan and heat sink on the LED prevented detectable heat from reaching the skin of the patient. Patients were asked if they could tell if they had just received a treatment with the light on or off, and all reported that they could not detect any differences between the treatments. After all 4 interventions were completed, the patients were asked about adverse physical or psychological symptoms.

Two weeks and 4-weeks after the treatment day, each patient was given a follow-up HAM-D, HAM-A, PANAS, and the side-effects questionnaire.

### Statistics

Our primary outcomes were changes from baseline in HAM-D and HAM-A scores at 2 weeks and 4 weeks post-treatment, and our secondary outcomes were change in PANAS score at 2 weeks and 4 weeks post-treatment, difference in immediate after treatment in PANAS score between NIR on and NIR off, and difference in rCBF between NIR on and NIR off. We also tested for associations between treatment side that was matched or unmatched with the hemisphere with a positive HEV and PANAS changes immediately following treatment. Here we used hierarchical linear models with treatment (NIR on or off), side of treatment (F3 or F4), and their interaction as predictors. HEV and its interaction with the side of treatment were added to these models to test for an association between these factors and immediate treatment benefit.

To test for changes in symptom ratings at 2 weeks and 4 weeks post-treatment, we used repeated measures linear regression models with measurement time as a categorical predictor and unstructured covariance between repeated measurements. Paired comparisons between measurement times were conducted in the presence of an overall difference among mean symptom ratings at baseline, 2 weeks, and 4 weeks significant at the alpha = 0.05 level. To facilitate the clinical interpretation of our findings and comparison with other studies, we also report the number of participants who were "improvers" (20% or more decrease from baseline) and "responders" (50% or more decrease from baseline) based on HAM-D and HAM-A scores, the number who achieved "remission" (a score less than 8 or 11) based on HAM-D and HAM-A scores, and the mean ± standard deviation percentage change at 2 weeks and 4 weeks for the three clinical measures.

To test for associations between treatment and rCBF, paired t-tests compared average rCBF across the left and right hemispheres and rCBF within each hemisphere between NIR on and NIR off. To test whether any treatment effect differed between the left and right hemispheres, an additional paired t-test compared mean differences in rCBF between NIR on and NIR off between the left and right hemispheres. We also considered hierarchical models for the association between treatment and rCBF, but the data did not support their complexity.

Post-hoc tests for associations among hemispheric valence, differences in rCBF, and two-week changes in HAM-A and HAM-D scores were conducted using linear regression with random intercepts for subjects when appropriate. Both point changes and percentage changes in HAM-D and HAM-A were considered as outcomes. These models treated hemispheric valence as a quantitative variable.

Statistical significance required two-tailed p-values less than 0.05. A Bonferroni correction was applied to results from the models for changes in HAM-D and HAM-A scores with treatment to account for our choice of two primary clinical outcomes. Other results were not adjusted for multiple comparisons. Statistical analyses were conducted using R statistical software (version 2.9.2) and the PROC MIXED routine for SAS statistical software (version 9.1.3, Cary, NC).

## Results

### Changes in rCBF in response to PBM with NIR, comparing the light off and light on conditions

**Figure 2:**
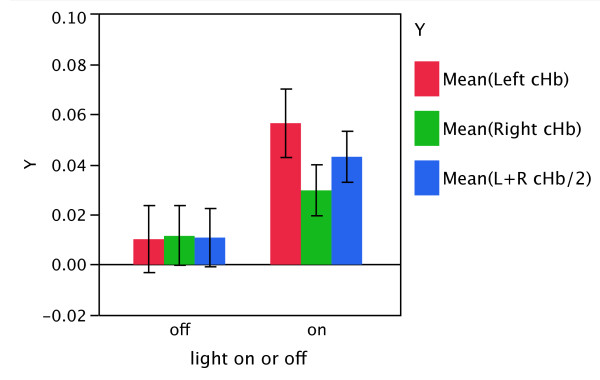
**Pre-frontal blood flow, "NIR on" versus "placebo"**. A comparison of the mean left-, right-sided, and left + right pre-frontal total hemoglobin (cHb) measurements (arbitrary, relative units) recorded during the two 4-minuted NIR treatments (F3 and F4), light on conditions, and during the two 4-minute placebo (LED off) conditions (F3 and F4). cHb is an index of regional cerebral blood flow (rCBF). Mean rCBf across hemispheres (left + right/2) increased from 0.011 units in the sham condition to 0.043 units in the treatment condition, for a difference of 0.032 (95% CI: -0.016, 0.080) units, though this result did not reach statistical significance (t_9 _= 1.52, p = 0.16). The increase with treatment was 0.046 (95% CI: -0.004, 0.097; t_9 _= 2.07, p = 0.07) units in the left hemisphere and 0.018 (95% CI: -0.033, 0.069; t_9 _= 0.80, p = 0.44) units in the right hemisphere, but the difference between hemispheres was also not statistically significant (95% CI for difference: -0.01, 0.063; t_9 _= 1.83; p = 0.10). Error bars represent 1 standard error from the mean.

Mean rCBf across hemispheres (left + right/2) increased from 0.011 units in the sham condition to 0.043 units in the treatment condition, for a difference of 0.032 (95% CI: -0.016, 0.080) units, though this result did not reach statistical significance (t_9 _= 1.52, p = 0.16). The increase with treatment was 0.046 (95% CI: -0.004, 0.097; t_9 _= 2.07, p = 0.07) units in the left hemisphere and 0.018 (95% CI: -0.033, 0.069; t_9 _= 0.80, p = 0.44) units in the right hemisphere, but the difference between hemispheres was also not statistically significant (95% CI for difference: -0.01, 0.063; t_9 _= 1.83; p = 0.10). Figure 2 illustrates these results.

### Immediate affect responses to NIR or sham

There were no significant differences in PANAS scores (immediately after treatment) between the NIR on and off treatment conditions (t_19 _= -1.47, p = 0.16).

Although the PANAS scores were not significantly different with the light on or off, we wondered whether applying NIR-PBM to a hemisphere with a positive HEV would elicit more positive affect than when it was applied to a hemisphere with a negative HEV. We found a statistically significant interaction between NIR on treatment and HEV supporting this hypothesis (t_18 _= 2.23, p = 0.04). When NIR was applied to the HEV-positive hemisphere, mean PANAS scores improved relative to when it was applied to the HEV-negative hemisphere. The more matched the treatment side and side of more positive HEV, the greater the benefit of the infrared treatment condition relative to the sham condition, where the most pronounced difference between treatments was a disadvantage of treatment relative to sham for unmatched HEV.

### Two week and 4-week psychological measures

All 3 of our post-treatment outcome measures showed improvements at 2 weeks, which remained but were attenuated at 4 weeks. The improvement was statistically significant for HAM-D and HAM-A but not for PANAS.

#### Hamilton depression rating scale

Evaluating our first primary outcome measure, the HAM-D, at 2 and 4-weeks post treatment (to both F3 and F4), we found that there were significant changes in HAM-D following treatment (F_2,8 _= 14.98, p = 0.004), with the lowest symptom scores occurring 2 weeks post-treatment. Mean HAM-D decreased significantly by 13.20 (95% CI: 6.46-19.94) points at 2 weeks (t_9 _= -5.26, p = 0.001) and 6.50 (95% CI: 0.28-12.72) points at 4 weeks (t_9 _= -2.81, p = 0.04). The increases in HAM-D between 2 weeks and 4 weeks were also significant (t_9 _= 4.45, p = 0.003).

**Figure 3:**
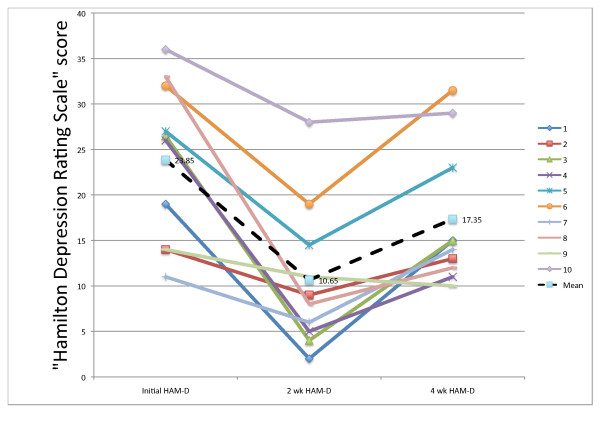
**Initial, 2-week, and 4-week HAM-D scores**. The individual patient's Hamilton Depression Rating Scores at Baseline, 2-weeks, and at 4-weeks. A high score suggests more depression. Fifteen or above is suggestive of a clinical depression and below 8 is suggestive of a remission. The legend numbers correspond to the patient numbers. The mean scores are indicated.

Mean percentage reductions in HAM-D scores were 54.3% ± 26.1 at 2 weeks post-treatment and 23.0% ± 27.1 at 4-weeks post-treatment. At 2-weeks all 10 patients were "improvers," defined in the literature as those patients who respond to an intervention for depression with at least a 20% reduction in HAM-D; 4 out of 10 patients were "responders" (>50% reduction in HAM-D), among whom there was a reduction of 82.8% ± 5.8. Four out of the 10 patients achieved "remission," (HAM-D <8). Some authors define "remission" as a score ≤ 10 [43], and using that standard, 6 out of 10 achieved "remission." At 4 weeks, we observed that 5 out of 10 patients were still "improvers;" 2 were still "responders," and no patient still achieved "remission" at <8, but one achieved "remission" at the ≤ 10 criterion. Figure 3 shows the HAM-D results for the 10 individual patients at 2-weeks post-treatment. At that point, 4 of the 5 males, but no females achieved a remission at <8.

#### Hamilton anxiety rating scale

Evaluating our second primary outcome measure, the HAM-A, at 2- and 4-weeks post-treatment, we found that there were significant changes in HAM-A following treatment (F_2,8 _= 11.70, p = 0.008), with the lowest symptom scores occurring 2-weeks post-treatment. Mean HAM-A decreased significantly by 14.90 (95% CI: 6.77-23.03) points at 2 weeks (t_9 _= -4.92, p = 0.002) and 9.00 (95% CI 2.66-15.34) points at 4 weeks (t_9 _= -3.81, p = 0.008). The increases in HAM-A between 2 weeks and 4 weeks were also significant (t_9 _= 4.35, p = 0.004).

**Figure 4:**
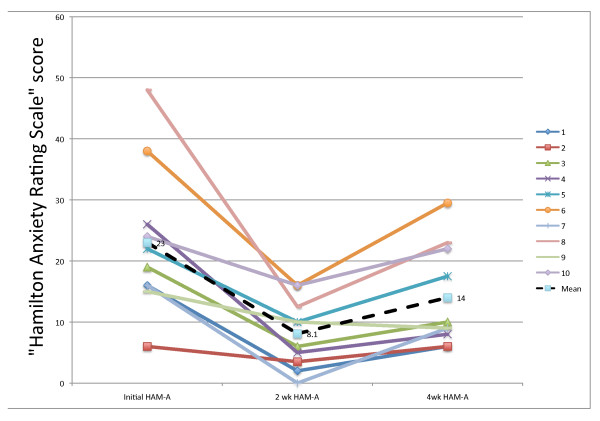
**Initial, 2-week, and 4-week HAM-A scores**. The individual patient's Hamilton Anxiety Rating Scores at Baseline, 2-weeks, and at 4-weeks. A high score suggests more anxiety. Fifteen or above is suggestive of a clinical anxiety disorder and below 8 is suggestive of a remission. The legend numbers correspond to the patient numbers. The mean scores are indicated.

Mean percentage reductions in HAM-A scores were 63.1% ± 23.0 at 2 weeks post-treatment and 36.6% ± 23.0 at 4-weeks post-treatment. At 2 weeks, all 10 patients were "improvers;" 7 out of 10 were "responders" (>50% reduction in HAM-A), among whom there was a reduction of 74.7% ± 16.2. Five of the 10 patients achieved "remission" at HAM-A <8, and 7 of the 10 achieved it at HAM-A ≤ 10. At 4-weeks, 9 of the 10 were still "improvers," 3 of the 10 were still "responders," and 2 of the 10 still achieved "remission" at HAM-A <8, and 6 of the 10 achieved a remission at HAM-A ≤ 10. Figure 4 shows the HAM-A results for the 10 individual patients at 2-weeks post-treatment. At that point, 4 of the 5 males achieved a remission at <8, as did 1 of 5 females. Table 4. summarizes the HAM-D and the HAM-A outcomes at 2- and 4-weeks post-treatment.

**Table 4:** Patient outcomes at 2- and at 4-weeks post-treatment on the HAM-D and the HAM-A. Data are means ± sd

	Measure	Initial minus post-treatment	% Decrease	% Decrease >20%	% Decrease >50%	Score <8	Score ≤ 10
				**Improvers**	**Responders**	**Remission**	**Remission**

2 weeks post-treatment	HAM-D	13.2 ± 7.9	54.3% ± 26.1	100%	40%	40%	60%
	HAM-A	14.9 ± 9.6	63.1% ± 23.0	100%	70%	50%	70%

4 weeks post-treatment	HAM-D	6.5 ± 7.3	23.0% ± 27.1	50%	20%	0.0	10%
	HAM-A	9.0 ± 7.5	36.6% ± 23.0	90%	30%	20%	60%

Statistical tests are presented in the text.

#### PANAS scale

The mean initial PANAS was 3.9 ± 5.5; the 2-week mean PANAS was 8.8 ± 6.6; and the 4-week mean PANAS was 6.8 ± 4.9. Unlike the HAM-D and HAM-A, an increase in the PANAS score represents an improvement. All 3 of our outcome measures showed an improvement at 2 weeks, which decreased by 4 weeks but remained better than baseline. Mean PANAS increased by 4.90 (95% CI: 0.49-9.31) points at week 2 relative to baseline and 2.85 (95% CI: -1.28-6.98) points at week 4 relative to baseline. These changes in PANAS scores at 2- and 4-weeks post-treatment did not achieve statistical significance (F_2,8 _= 3.21, p = 0.09). We observed an improvement in the PANAS scale from baseline to 2 weeks of 122% ± 183 and 54% ± 133 at 4 weeks.

### Predictors of 2-week outcomes

When percentage change in HAM-A (but not HAM-D) at 2 weeks was considered, there was a significant association between the left minus the right frontal rCBF and the baseline HEV score and the change in HAM-A such that a greater right positive HEV was associated with a greater rCBF in the right frontal pole and a greater left positive HEV was associated with a greater rCBF in the left frontal pole. This greater rCBF in the direction of positive hemispheric valence was associated with greater reductions in HAM-A scores (t_6 _= 3.26, p = 0.02), though there was no such association with differences in differential blood flow between the NIR on and NIR off conditions (t_6 _= 1.67, p = 0.15). When the differences between baseline and 2-week values for HAM-D or HAM-A were used, instead of the percent change, as the outcome measures, then there were no significant associations between the direction of rCBF and HEV (t_6 _= 0.40, p = 0.70 HAM-D; t_6 _= 0.12, p = 0.91 HAM-A)

### Safety

No adverse events or side effects were found after detailed questioning of the patients immediately after the initial visit to the laboratory and at 2 and 4 weeks post-treatment.

## Discussion

Many studies have reported beneficial effects from the application of red and NIR light to the body [44-46], and one group [17,18] reported beneficial effects applying transcranial NIR to the brain in stroke patients. We embarked on this study to see if the psychological status of patients with depression might benefit from the application of NIR light to the head. Although we recruited for patients with depression, we found that 9 of those who responded also manifest an anxiety disorder by SCID, including 3 who met criteria for both generalized anxiety disorder and PTSD. Seven of these patients had also a past history of opiate abuse, 6 treated with buprenorphine and 1 with methadone. We intended this as a small pilot study for an initial evaluation of our treatment's safety (immediately and over 4-weeks) and to look for indications that it might have some efficacy immediately after each treatment, and/or at 2 and at 4-weeks post-treatment. We had 4 treatment conditions, NIR at F3 and at F4, and "no light" with the mushroom fan on at F3 and F4, as placebo conditions. We measured also rCBF by NIRS to a depth of at least 1 cm at the left and right frontal poles of the brain to see if the NIR treatment might have a definite physiological effect, and, if so, to see if the blood flow measurements might shed any information about the treatment's mechanism of action. We measured the patients' baseline HEVs because our prior studies determined that the measurement might be useful in data analysis and in guiding treatment [39,41,42].

Our results showed that with one 4-minute NIR treatment on each side of the head there were marked benefits in both of our primary outcome measures, the HAM-D and the HAM-A. We observed the greatest benefit at 2-weeks post-treatment for both measures. At 4 weeks both showed a statistically significant improvement over baseline but a significant decline from 2-week levels. These results should be interpreted with caution since this was not a placebo-controlled trial.

The HAM-D and HAM-A are not suited for measuring immediate effects, but are used to measure outcomes over a longer period. The PANAS was most useful for evaluating immediate post-treatment effects, but was also used at 2 and 4 weeks. There were no statistically significant associations between treatment and PANAS scores, either at the time of treatment or during the four-week follow-up period.

The size of our sample was too small to represent the larger patient populations, but still within this population, a single, brief treatment with transcranial NIR light had effects that seem to compare well with other modalities. For example in a previous study we reported [42], using transcranial magnetic stimulation, to treat 37 refractory depressed patients over two weeks, and used the same 21-item HAM-D as in the present study, and found at 2-weeks following the completion of the treatment a mean percent decrease in HAM-D of 29.4% ± 26.1. In the present study, we found a mean percent decrease in the HAM-D of 54.3% ± 26.1 at 2-weeks post-treatment. The rTMS study had a larger population (N = 37), and the mean baseline HAM-D was 29.6 ± 5.6, which was higher than that for the population in this study (23.8 ± 8.8). Therefore, the studies cannot be directly compared, but it is unusual for to find two studies with the identical outcome measures applied at identical times. In a recent rTMS study by Stern et al [43] in which they compared outcomes using different stimulation parameters, the best group had a remission (≤ 10) rate of 40% at 2-weeks post-treatment; in the present study there was a remission rate of 60% at 2-weeks.

Gershon et al [47] reviewed the efficacy of rTMS and found a wide range of " % responders" (decrease % in HAM-D ≥ 50%) from 10 to 49% among 5 sham controlled studies. In all of these studies the active treatment was far superior to the sham, which ranged from a 0% to a 25% response rate. Loo et al [48] reported in a recent rTMS study, using twice daily left-sided high frequency rTMS, a decrease in HAM-D of 38.5% immediately after a 2-week sham controlled study, compared to 54.3% in our study 2-weeks after treatment.

Some studies have compared rTMS with electroconvulsive therapy (ECT) [49-51], and found them generally to have a similar efficacy in severe depression. For instance, Janicak et al [50] compared up to 20 rTMS treatments with 3 to 12 ECT treatments and reported at the end of treatment the rTMS group had a "remission" rate (<8) of 46% compared with 56% for the ECT group. The authors did not report 2-week post-treatment results. Our 2-week post-treatment HAM-D scores indicated that 40% had achieved "remission" (< 8).

In a recent study comparing the efficacy of 6 right-sided ECT treatments with 6 bilateral over 3 weeks, Eschweiler et al. [52] found that both groups had a 37% decrease in HAM-D at the end of treatment. Each group had 26% "responders" (≥ 50%) at the end of treatment. Again, our group at 2-weeks post-treatment had a mean decrease in HAM-D of 54.3% with 40% "responders" (≥ 50%).

In a recent study, Tadi et al [53] compared the outcomes in HAM-D from baseline to 10 weeks for 223 patients with depression randomized between 4 treatment groups: sertraline, placebo pill, cognitive-behavioral therapy (CBT), and guided self-help group (GSG). At 10-weeks, the completion of the treatments, 44% of the sertraline group responded (HAM-D % decrease ≥ 50%), compared with 19% for the placebo group, 20% for the CBT group, and 19% for the GSG group. By the second week 49% of the sertraline group, 40% of the placebo group, 39% of the CBT group, and 35% of the GSG group showed improvement defined as a decrease in HAM-D of ≥ 20%. In our study at 2-weeks post-treatment 100% showed improvement (decrease in HAM-D ≥ 20%).

Katz et al [54] reported HAM-D outcomes for 70 depressed patients (baseline HAM-D = 23.5) randomly divided between 3 treatment groups: desipramine, paroxetine, and placebo. At 2-weeks the desipramine group had a mean % decrease in HAM-D of 45%, the paroxetine group, 24%, and the placebo group, 36%.

Bech et al [55] performed a meta-analysis of 16 US trials involving depressed patients comparing fluoxetine with either tricyclic antidepressants or with placebo in trials of at least 6-weeks. The authors reported that among the 1914 patients intended to treat with fluoxetine, 38.5% were responders (HAM-D reduction ≥ 50%), while among the 686 TCA treated patients this measure was 35.5%, and among the 847 placebo treated patients the measure was 24.2%.

In regard to anxiety, Leichsenring et al [56] found that 29 patients with a generalized anxiety disorder (GAD) treated with CBT for 30 weeks achieved a 50.7% reduction on the HAM-A at the end of treatment, and that 28 patients treated with short-term psychodynamic psychotherapy over the same time period achieved a 42.8% reduction. In our study at 2-weeks post-treatment, our patients achieved a reduction in HAM-A of 63.1%. Moreover, Montgomery et al [57] reported pooled data from 6 double-blinded, placebo-controlled, 4 to 6-week trials for patients with GAD treated with a benzodiazepine (either alprazolam or lorazepam), with pregabalin (PGB), or with placebo. The benzodiazepine group had a mean decrease in HAM-A from baseline to the end of treatment of -11.0 points, the PGB group had a decrease of -11.2, and the placebo group, -8.3. In our study, we found a decrease in HAM-A from baseline to 2-weeks post-treatment of 14.9 points.

Even though our 2-week results compare well with the other reported treatments cited above, the 2 and 4-week outcomes were unblinded, and did not have a placebo control. Further, comparisons between treatments need to be made with a single study and those results replicated.

Some of our secondary experiments showed results in support of our initial hypotheses. For example, there was greater rCBF during NIR on versus off, although this difference did not achieve statistical significance. NIR on was more successful relative to NIR off when treatment was applied to a hemisphere with more positive HEV.

An increase in rCBF with NIR is consistent with an effect of NIR treatment on the brain. This effect on the brain (whatever its complex nature) likely relates to the alterations in affect. Together with our result that immediate psychological benefit of infrared treatment was associated with positive HEV, that the 2-week HAM-A outcomes related to the HEV value and left - right rCBF is consistent with the hypothesis we presented at length in a previous publication [39], stating that the right hemisphere is often associated (unexpectedly) with a positive HEV and that knowing a patient's HEV can enlighten data reduction and possibly guide treatment. We did not use HEV to guide therapy in this study, but we think that future studies should consider this possibility. In two other previous, independent publications we reported that HEV by LVFS could predict positive responses to left-sided rTMS [41,42]. While promising, the result of an association with 2-week changes in HAM-A should be interpreted with caution since it was based on a post-hoc analysis, was sensitive to our quantification of change in HAM-A (percent vs. points change), and did not hold for HAM-D.

Because this is the first trial applying NIR to the brain, we wanted to be extremely vigilant for negative side effects. We found none, during or after the procedure. During the treatments we turned off the fluorescent lights to prevent interference with our NIRS data and the fan created a drone and light breeze, all of which seemed to relax the patients, although we did not formally measure this. Certainly, no patient complained of headaches or any other physical discomfort. No patient dropped out of the study and all continued through the 4-week follow-up. Six patients spontaneously reported feeling much improved at 2-weeks and attributed that improvement (rightly or wrongly) to the treatment. The other 4 patients did not feel any effect, positive or negative, from the treatment, including one man who had an 85% improvement on his HAM-D and a 68% improvement on his HAM-A at 2-weeks. Thus, we observed the treatment to be comfortable, pleasant, easy to apply, and safe.

The mechanism by which NIR-PBM has improved mood is not understood. PBM is known to improve blood flow in skin (as measured by laser Doppler) [58]. The fact that HEV may play a role in the response suggests that positive neural circuits might somehow be stimulated by NIR light or negative neural circuits may be inhibited. NIR is known to increase mitochondrial ATP and nerve growth factors. We feel that our outcome findings must be replicated in double blind, randomized, placebo-controlled prospective outcome studies with large numbers and various populations. The method of treatment should also be studied to attempt to optimize the results. Some possibilities are to use pulsed light, try different anatomical locations, different treatment schedules (daily, weekly, biweekly, etc) as well as different light wavelengths and total energy densities. We would like to study also whether HEV should guide treatment. Lastly, this treatment might benefit from a possible synergy with other treatments such as psychotherapy and psychotropic medications. All the subjects in this study remained on their usual treatment and no one altered their usual treatment during this study. If further study confirms our results or improves upon them, then an intense search for the mechanism of action will be highly desirable and might lead to greater knowledge of mind-brain interactions, the psychophysiology of mental states, including the effects of trauma, and of treatment benefits.

## Conclusion

We gave one 8-minute treatment with NIR-PBM to 10 patients with major depression, including 7 with a history of substance abuse (6 with a past history of opiate abuse and one with a past history of alcoholism), and 9 with an anxiety disorder, including 3 with PTSD. We found significant reductions in both mean HAM-D and HAM-A rating at 2 and 4 weeks following treatment. At 2-weeks post treatment 6 of 10 of patients had a remission (a score ≤ 10) on the HAM-D and 7 of 10 on the HAM-A. We observed no side effects. This small feasibility study suggests that follow-up double blind randomized placebo-controlled trials of NIR-PBM for the treatment of psychological disorders are indicated.

## Competing interests

FS, on August 14, 2009, filed an application for a US patent covering the subject matter of this paper. He has no other financial or non-financial competing interests related to this manuscript. The other authors declare that they have no competing interests.

## Authors' contributions

FS conceived of the study and contributed to its design, coordination, acquisition and analysis and interpretation of data, and drafting of the manuscript; ALJ participated in the coordination of the study and in the acquisition of data; CR performed the statistical analyses and participated in the revision of the manuscript; AP participated in the design of the study; MHT participated in the design of the study and in the revision of the manuscript; RHW guided the implementation of the LED technology used in the study and contributed to the drafting of the manuscript; MRH guided the photomedicine theoretical and practical aspects of the study and participated in the drafting of the manuscript. All authors read and approved the final manuscript.
